# The association between early-life famine exposure and adulthood risk of thyroid diseases

**DOI:** 10.3389/fnut.2025.1633077

**Published:** 2025-09-11

**Authors:** Li Liu, Liliang Yu, Daikun Zheng, Dongmei Zhang, Lijun Liu, Li Wan, Yan Shen, Hongfeng Cheng

**Affiliations:** ^1^Chongqing Three Gorges Medical College, Chongqing, China; ^2^Department of Breast and Thyroid Surgery, The First Affiliated Hospital of Chongqing Medical University, Chongqing, China; ^3^Department of Ultrasonography, The Third Affiliated Hospital of Chongqing Medical University, Chongqing, China; ^4^Health Management Center, The Second Affiliated Hospital, Chongqing Medical University, Chongqing, China

**Keywords:** Chinese Great Famine, thyroid disease, thyroid function, fetal period, childhood

## Abstract

**Background:**

Early-life exposure to famine is associated with an increased risk of various metabolic disorders. Nevertheless, evidence regarding its long-term effects on thyroid function and disease risk in older adulthood remains scarce. This study investigates the impact of fetal and childhood exposure to the Chinese Great Famine (1959–1961) on thyroid function and disorders in late life.

**Methods:**

This cross-sectional study enrolled 1,956 participants who completed health examinations at a public hospital-based Physical Examination Center in Chongqing between 2022 and 2023. Based on birth cohorts, participants were stratified into three groups: the unexposed group (individuals born in 1963.1.1–1967.12.31), the fetal-exposed group (individuals born in 1959.1.1–1962.12.31), and the childhood-exposed group (individuals born in 1949.1.1–1958.12.31). Binary logistic regression models were used to evaluate the association between famine exposure and thyroid disease risk in later life. Multiple linear regression analyses compared thyroid function biomarkers between famine-exposed and non-exposed groups, adjusting for potential confounders.

**Results:**

In this study, 373 participants (19.1%) were exposed to the Chinese Great Famine during the fetal period, with 597 individuals (30.5%) experiencing childhood exposure. After adjusting for gender, smoking history, drinking history, dietary salt preference, current exercise status, educational level, body mass index (BMI), the fetal-exposed group demonstrated significantly elevated risks of both overt hyperthyroidism [OR = 4.36, 95% CI (1.02–18.71)] and subclinical hyperthyroidism [OR = 3.13, 95% CI (1.03–9.51)] compared to the non-exposed group. After adjusting for multiple comparisons using the Benjamini-Hochberg FDR method, fetal famine exposure maintained a statistically significant inverse association with thyroid nodule risk relative to childhood exposure [OR = 0.69, 95% CI (0.51–0.93)]. No significant associations were observed between famine exposure and hypothyroidism, thyroid autoantibody positivity, or autoimmune thyroid disorders. Notably, childhood-exposed participants exhibited higher thyroglobulin antibody (TgAb) levels versus non-exposed individuals [*β* = 40.30, 95% CI (2.21–78.40)].

**Conclusion:**

Fetal exposure to the Chinese Great Famine reduced thyroid nodule risk whereas childhood exposure increased TgAb levels, revealing distinct developmental windows for nutritional programming of thyroid health. These findings underscore the importance of timing in malnutrition-related thyroid dysfunction.

## Background

Thyroid disorders, ranking among the most prevalent endocrine disorders worldwide, affect over 200 million individuals globally (1). Thyroid dysfunction disrupts physiological metabolic processes and demonstrates significant associations with adverse health outcomes including cardiovascular morbidity, cerebrovascular events, and psychological disturbances (2–4). These conditions have emerged as a critical global health priority. Consequently, identifying modifiable risk factors for thyroid dysfunction is essential for advancing global health outcomes.

Early-life environmental exposures, particularly nutritional status, exert profound and enduring impacts on subsequent health trajectories (5–7). For instance, infants born to mothers with anemia and malnutrition exhibit reduced triiodothyronine (T3) levels (8), while women with lower birth weight and smaller body size at birth demonstrate elevated risks of hypothyroidism in adulthood (9). As an extreme environmental stressor, famine may induce developmental programming of physiological and metabolic systems during critical early-life windows, with lifelong health consequences. Existing studies reveal that early-life famine exposure significantly increases risks of aging-related outcomes (10, 11), fractures (12, 13), obesity (14, 15), diabetes mellitus (16, 17), and chronic kidney disease (18, 19) in later life. However, evidence regarding the impact of early famine exposure on adult thyroid regulation remains limited.

The Dutch Hunger Winter cohort study (20) found no statistically significant association between fetal famine exposure and thyroid disease incidence at age 50, though exposed individuals exhibited significantly lower thyroid stimulating hormone (TSH) levels compared to non-exposed counterparts. Conversely, research on Chinese famine survivors (21) revealed that fetal-exposed individuals had significantly higher adult TSH levels than non-exposed controls. Given these conflicting findings, this study systematically evaluates the long-term consequences of prenatal and childhood famine exposure on thyroid homeostasis and disorder susceptibility in late adulthood.

## Methods

### Study population

This cross-sectional study utilized data from the Physical Examination Center of a public hospital in Chongqing, China, enrolling singleton live births delivered at full term (gestational age ≥37 weeks) between January 1, 1949, and December 31, 1967. Participants with incomplete thyroid function tests, thyroid ultrasonography records, or anthropometric data were excluded, resulting in a final analytical cohort of 1,956 individuals. The core period of the Chinese Great Famine was 1959–1961, but the exact end time of famine conditions varied across regions. With reference to previous studies on the Chinese famine (22), participants were stratified into three groups based on birth dates and famine exposure periods: (1) the non-exposed group (born January 1, 1963–December 31, 1967), (2) the fetal-exposed group (born January 1, 1959–December 31, 1962, in utero during the famine peak), and (3) the childhood-exposed group (born January 1, 1949–December 31, 1958, aged 1–10 years during the famine). To minimize age-related confounding effects, an age-balanced comparison group was constructed by merging the non-exposed and childhood-exposed groups. This study was approved by the human research ethics committee of the Second Affiliated Hospital of Chongqing Medical University.

### Definition of thyroid disorders

Fasting blood samples were collected from all participants in the morning following a standardized 10-h overnight fast. Thyroid function tests included assessment of TSH, free triiodothyronine (FT3), free thyroxine (FT4), and antibodies against thyroid peroxidase (TPO) and thyroglobulin (TG). The serum levels of FT3, FT4, TSH, thyroid peroxidase antibodies (TPOAb), and thyroglobulin antibodies (TgAb) were detected by the Cobas 601 analyzer (Roche Diagnostic, Switzerland). Thyroid nodules were defined as one or more nodules (>5 mm) without goiter on B-mode ultrasonography.

The diagnostic criteria for thyroid dysfunction were provided by the detection kit manufacturer. Overt hyperthyroidism is defined as TSH < 0.27 mIU/L and FT4 > 22.0 pmol/L or FT3 > 6.8 pmol/L. Subclinical hyperthyroidism is defined as TSH < 0.27 mIU/L with FT4 and FT3 within the normal range (FT4 between 12.0 and 22.0 pmol/L; FT3 between 3.1 and 6.8 pmol/L). Overt hypothyroidism is defined as TSH > 4.2 mIU/L and FT4 < 12.0 pmol/L. Subclinical hypothyroidism is defined as TSH > 4.2 mIU/L, FT4 between 12.0 and 22.0 pmol/L, positive TPOAb as TPOAb > 34 IU/mL, and positive TgAb as TgAb > 115 IU/mL. Autoimmune thyroiditis (AIT) is defined as TPOAb > 34 IU/mL or TgAb > 115 IU/mL.

### Definition of the covariates

Demographic characteristics, including birth date, sex, smoking history, drinking history, dietary salt preference, current exercise status, and education level, were measured by self-reports. Smoking history includes those who have smoked in the past or are currently smoking. Drinking history includes subjects who are drinking alcohol and those who have stopped drinking alcohol. Dietary salt preference was divided into three categories: salty taste, light taste and no preference. The current exercise status is defined as regular exercise, occasional exercise and no exercise. Education levels were categorized as junior school and below and high school or above. During the health examination, anthropometric indices, including height, weight, waist circumference (WC), and hip circumference (HC), were measured by trained health workers according to standard protocols. Body mass index (BMI) was calculated based on body weight (kg) and height (m). The waist-to-hip ratio (WHR) is calculated by dividing the WC (cm) by the HC (cm).

### Statistical analysis

All statistical analyses were performed using R version 4.4.1. A two-sided *p* < 0.05 was considered statistically significant. Continuous variables were represented as mean ± standard deviation or median (percentiles) based on whether they follow a normal distribution. Differences among groups were evaluated using one-way analysis of variance (ANOVA) or non-parametric tests, depending on the normality of the data distribution. Categorical variables were expressed as frequencies (percentages), and the chi-square test or Fisher’s exact test is utilized to compare the differences between groups. A binary logistic regression model was used to clarify the relationship between famine exposure and thyroid diseases. A multiple linear regression analysis was employed to estimate the levels of TSH, TgAb, and TPOAb between the exposed group and the non-exposed group.

## Results

Table 1 presents the demographic and clinical characteristics of the study population, consisting of 1,008 women (52%) and 948 men (48%). Among participants, 373 (19%) experienced fetal exposure to the Chinese Great Famine, 597 (31%) had childhood exposure, and 986 (50%) were unexposed. As shown in Table 1, compared with the unexposed group, those who were exposed to famine during childhood or the fetal stage had a higher proportion of females, less smoking history, and a lower educational level. However, there were no significant differences in other indicators such as drinking history, dietary salt preference, current exercise status, BMI, WHR (*p* > 0.05). The overall prevalence of thyroid diseases among the participants was as follows: overt hyperthyroidism 1% (14 cases), subclinical hyperthyroidism 1% (19 cases), overt hypothyroidism 1% (18 cases), subclinical hypothyroidism 18% (351 cases), positive TgAb 4% (75 cases), positive TPOAb 10% (203 cases), AIT 14% (278 cases), and thyroid nodules 72% (1,402 cases). The levels of TSH, TgAb and TPOAb were higher in the fetal exposure group than in the non-exposure group, yet there was no statistical significance.

**Table 1:** Baseline characteristics.

Characteristics	Non exposed	Fetal-exposed	Childhood-exposed	*p*-value
Birth date	1963.1.1–1967.12.31	1959.1.1–1962.12.31	1949.1.1–1958.12.31	
*N*	986	373	597	
Age (median [IQR], years)	58.56 (57.71, 59.27)	61.14 (60.35, 62.33)	67.02 (65.37, 68.77)	<0.001
Sex, *n* (%)				0.031
Women	481 (49)	210 (56)	317 (53)	
Men	505 (51)	163 (44)	280 (47)	
Education, *n* (%)				<0.001
Junior school and below	340 (34)	149 (40)	312 (52)	
High school and above	646 (66)	224 (60)	285 (48)	
Dietary salt preference, *n* (%)				0.808
Salty taste	55 (6)	22 (6)	37 (6)	
Light taste	86 (9)	26 (7)	45 (8)	
No preference	845 (86)	325 (87)	515 (86)	
Smoking history, *n* (%)	221 (22)	66 (18)	83 (14)	<0.001
Drinking history, *n* (%)	58 (6)	20 (5)	28 (5)	0.596
Current exercise status, *n* (%)				0.072
No exercise	393 (40)	161 (43)	272 (46)	
Exercise occasionally	295 (30)	110 (29)	144 (24)	
Exercise regularly	298 (30)	102 (27)	181 (30)	
BMI (median [IQR], kg/m^2^)	23.99 (22.15, 25.94)	23.95 (22.21, 25.81)	23.88 (22.08, 25.78)	0.821
WHR (median [IQR])	0.88 (0.83, 0.93)	0.88 (0.82, 0.93)	0.88 (0.83, 0.92)	0.981
Overt hyperthyroidism, *n* (%)	3 (0)	5 (1)	6 (1)	0.050
Subclinical hyperthyroidism, *n* (%)	6 (1)	7 (2)	6 (1)	0.102
Overt hypothyroidism, *n* (%)	9 (1)	2 (1)	7 (1)	0.633
Subclinical hypothyroidism, *n* (%)	161 (16)	69 (18)	121 (20)	0.134
TgAb positive, *n* (%)	31 (3)	18 (5)	26 (4)	0.258
TPOAb positive, *n* (%)	96 (10)	43 (12)	64 (11)	0.594
AIT, *n* (%)	127 (13)	61 (16)	90 (15)	0.202
Thyroid nodules, *n* (%)	689 (70)	260 (70)	453 (76)	0.024
FT3 (median [IQR], pmol/L)	4.40 (4.10, 4.80)	4.40 (4.10, 4.70)	4.40 (4.10, 4.70)	0.130
FT4 (median [IQR], pmol/L)	16.10 (14.70, 17.30)	16.00 (14.90, 17.50)	16.00 (14.70, 17.30)	0.662
TSH (median [IQR], mIU/L)	2.54 (1.55, 3.62)	2.63 (1.48, 3.89)	2.63 (1.65, 3.90)	0.159
TgAb (median [IQR], IU/mL)	18.80 (10.00, 40.45)	20.70 (10.00, 73.90)	19.30 (10.00, 74.80)	0.239
TPOAb (median [IQR], IU/mL)	5.00 (5.00, 16.17)	6.30 (5.00, 22.00)	6.10 (5.00, 20.40)	0.128

IQR, interquartile range; BMI, body mass index; WHR, waist-to-hip ratio; TgAb, thyroglobulin antibodies; TPOAb, thyroid peroxidase antibodies; AIT, autoimmune thyroiditis; FT3, free triiodothyronine; FT4, free thyroxine; TSH, thyroid stimulating hormone.

Table 2 shows the relationship between fetal famine exposure and the risk of thyroid diseases using different control groups. After adjusting for gender, smoking history, drinking history, dietary salt preference, current exercise status, education level, and BMI, fetal-exposed individuals exhibited significantly elevated risks of overt hyperthyroidism [OR = 4.36, 95% CI (1.02–18.71)] and subclinical hyperthyroidism [OR = 3.13, 95% CI (1.03–9.51)] compared to the non-exposed group. No significant associations were observed for other thyroid outcomes, including overt hypothyroidism [OR = 0.51, 95% CI (0.11–2.39)], subclinical hypothyroidism [OR = 1.09, 95% CI (0.79–1.49)], TgAb positivity [OR = 1.40, 95% CI (0.77–2.56)], TPOAb positivity [OR = 1.11, 95% CI (0.75–1.64)], AIT [OR = 1.21, 95% CI (0.86–1.70)], or thyroid nodules [OR = 0.97, 95% CI (0.74–1.26)] (*p* > 0.05 for all). Compared with the age-balanced group, the risk of subclinical hyperthyroidism in the fetal exposure group increased significantly [OR = 3.26, 95% CI (1.16–9.15)]. After adjusting for multiple comparisons using the Benjamini-Hochberg false discovery rate (FDR) method, fetal famine exposure maintained a statistically significant inverse association with thyroid nodule risk relative to childhood exposure [OR = 0.69, 95% CI (0.51–0.93)].

**Table 2:** The associations between fetal-exposed group and thyroid disorders with different control groups.

Fetal-exposed group No. of cases/sample size (%)	Control groups		OR [CI]	*P*
	Control groups	No. of cases/sample size (%)		
Overt hyperthyroidism 5/373(1.3)	Non-exposed	3/986(0.3)	4.36(1.02–18.71)	0.048
	Childhood-exposed	6/597(1.0)	0.95(0.28–3.22)	0.931
	Age-balanced	8/1344(0.6)	2.13(0.68–6.64)	0.195
Subclinical hyperthyroidism 7/373(1.9)	Non-exposed	6/986(0.6)	3.13(1.03–9.51)	0.044
	Childhood-exposed	6/597(1.0)	1.77(0.58–5.43)	0.317
	Age-balanced	8/1344(0.6)	3.26(1.16–9.15)	0.025
Overt hypothyroidism 2/373(0.5)	Non-exposed	9/986(0.9)	0.51(0.11–2.39)	0.392
	Childhood-exposed	7/597(1.2)	0.44(0.09–2.24)	0.323
	Age-balanced	12/1344(0.9)	0.53(0.12–2.42)	0.414
Subclinical hypothyroidism 69/373(18.5)	Non-exposed	161/986(16.3)	1.09(0.79–1.49)	0.610
	Childhood-exposed	121/597(20.3)	0.92(0.66–1.30)	0.648
	Age-balanced	235/1344(17.5)	1.02(0.75–1.38)	0.905
TgAb positive 18/373(4.8)	Non-exposed	31/986(3.1)	1.40(0.77–2.56)	0.271
	Childhood-exposed	26/597(4.4)	1.03(0.55–1.93)	0.937
	Age-balanced	50/1344(3.7)	1.22(0.70–2.14)	0.483
TPOAb positive 43/373(11.5)	Non-exposed	96/986(9.7)	1.11(0.75–1.64)	0.597
	Childhood-exposed	64/597(10.7)	1.10(0.72–1.67)	0.674
	Age-balanced	135/1344(10.0)	1.10(0.76–1.59)	0.620
AIT 61/373(16.4)	Non-exposed	127/986(12.9)	1.21(0.86–1.70)	0.281
	Childhood-exposed	90/597(15.1)	1.09(0.75–1.57)	0.660
	Age-balanced	185/1344(13.8)	1.15(0.83–1.59)	0.403
Thyroid nodules 260/373(69.7)	Non-exposed	689/986(69.9)	0.97(0.74–1.26)	0.802
	Childhood-exposed	453/597(75.9)	0.69(0.51–0.93)	0.016^*^
	Age-balanced	959/1344(71.4)	0.89(0.69–1.15)	0.359

All the analysis adjusted for sex, smoking history, drinking history, dietary salt preference, current exercise status, education level, and BMI.

No., numbers; CI, confidence interval; OR, odds ratio.

^*^FDR-adjusted *p* < 0.05.

Table 3 shows the multiple linear regression relationships among famine exposure and the levels of FT3, FT4, TSH, TgAb and TPOAb. Serum FT3 levels exhibited an inverse association with childhood famine exposure [*β* = −0.09, 95% CI (−0.17 – −0.01)]. This association remained significant after adjusting for covariates [*β* = −0.09, 95% CI (−0.17 – −0.01)]. Serum TgAb levels showed a positive association with childhood famine exposure [*β* = 46.58, 95% CI (8.84–84.33)]. This association was also significant after adjusting for covariates [*β* = 40.30, 95% CI (2.21–78.40)]. There were no significant differences in the levels of TSH [*β* = 0.49, 95% CI (−0.10–1.08)] or TPOAb [*β* = 7.58, 95% CI (−5.87–21.03)] between the childhood exposure group and the unexposed group. Similarly, fetal famine exposure showed no statistically significant effects on TSH [*β* = 0.21, 95% CI (−0.47–0.89)], TgAb [*β* = 30.83, 95% CI (−13.08–74.75)], or TPOAb [*β* = 7.97, 95% CI (−7.53–23.48)] levels compared to non-exposed individuals (*p* > 0.05 for all).

**Table 3:** Effects of fetal and childhood famine exposure on FT3, FT4, TSH, TgAb and TPOAb levels relative to non-exposed group.

Groups	Model	Fetal-exposed		Childhood-exposed	
		β [CI]	*p*	β [CI]	*p*
FT3 (pmol/L)	Model 1	-0.01(−0.11–0.08)	0.763	−0.09(−0.17--0.01)	0.030
	Model 2	−0.004(−0.10–0.09)	0.927	−0.09(−0.17--0.01)	0.021
FT4 (pmol/L)	Model 1	0.16(−0.11–0.44)	0.247	−0.02(−0.25–0.22)	0.891
	Model 2	0.18(−0.09–0.46)	0.189	0.04(−0.20–0.28)	0.740
TSH (mIU/L)	Model 1	0.31(−0.37–0.99)	0.375	0.56(−0.02–1.14)	0.060
	Model 2	0.21(−0.47–0.89)	0.542	0.49(−0.10–1.08)	0.101
TgAb (IU/mL)	Model 1	40.17(−4.08–84.42)	0.075	46.58(8.84–84.33)	0.016
	Model 2	30.83(−13.08–74.75)	0.169	40.30(2.21–78.40)	0.038
TPOAb (IU/mL)	Model 1	13.09(−2.76–28.94)	0.106	12.55(−0.97–26.08)	0.069
	Model 2	7.97(−7.53–23.48)	0.313	7.58(−5.87–21.03)	0.269

Model 1 unadjusted for any covariate.

Model 2 adjusted for sex, smoking history, drinking history, dietary salt preference, current exercise status, education level, and BMI.

## Discussion

This study demonstrates that compared to childhood famine exposure, fetal famine exposure was significantly associated with reduced risk of thyroid nodules in later life. After FDR correction for multiple comparisons, fetal exposure showed a suggestive but non-significant association with hyperthyroidism. Furthermore, we observed no significant association between early-life famine exposure and late-life TSH levels (*p >* 0.05). Notably, childhood famine exposure was inversely associated with FT3 levels and positively associated with elevated TgAb levels.

Previous studies suggest that the regulation of hypothalamic–pituitary-thyroid (HPT) axis function has a critical developmental window, during which early-life malnutrition may influence thyroid function in adulthood (23, 24). The fetal period represents a key developmental stage for HPT axis maturation. An animal study from Mexico demonstrated that nutritional deprivation during this stage could permanently alter thyroid hormone metabolism through epigenetic modifications (e.g., changes in deiodinase activity), leading to low FT4 and elevated TSH levels in adult rats (25). However, another animal study from Denmark found that late-gestational undernutrition resulted in hyperthyroidism in adult sheep (26), consistent with our findings. Large-scale human studies, including the Dutch Hunger Winter cohort (20) and Chinese famine research (21), reported no significant association between fetal famine exposure and overall thyroid disease incidence. Nevertheless, the Dutch cohort observed that mid-gestational famine exposure in females was associated with slightly reduced adult TSH levels and an increased risk of hyperthyroidism, suggesting potential programming effects of fetal famine exposure on HPT axis regulation. Our findings differ from previous studies in two key aspects: (1) we identified a trend toward increased hyperthyroidism risk with fetal famine exposure (though not statistically significant after FDR correction), and (2) we uniquely observed reduced thyroid nodule risk in the fetal-exposed group compared to childhood exposure. These discrepancies may stem from differences in diagnostic criteria (e.g., modern biochemical assays vs. historical clinical records) and variations in famine exposure duration across cohorts. Nevertheless, all studies, including ours, converge on a critical consensus: early-life famine exposure exerts lasting impacts on thyroid disease risk in later life, with specific manifestations shaped by developmental timing and post-famine environmental factors.

The Chinese Great Famine (1959–1961) was characterized by severe nationwide food shortages, resulting in extensive and profound nutritional deprivation. During this period, iodine—a critical trace element for thyroid hormone synthesis—was notably deficient, with significant implications for fetal thyroid development. Research indicates that insufficient maternal iodine intake during pregnancy directly impairs fetal thyroid hormone production, triggering compensatory upregulation of TSH via the HPT axis to meet metabolic demands (27). This compensatory mechanism may induce thyroid follicular hyperplasia and goiter formation. Notably, the widespread iodine deficiency during the famine starkly contrasts with China’s 1996 universal salt iodization (USI) policy, which shifted iodine intake from severe deficiency to sufficiency or even excess. Studies suggest that fetal thyroid structural alterations caused by iodine deficiency may lead to functional dysregulation in later high-iodine environments: hyperplastic thyroid tissues with enhanced iodine uptake capacity may overproduce thyroid hormones, increasing risks of subclinical or overt hyperthyroidism (28). Furthermore, the complexity of nutritional deprivation during the famine—including deficiencies in synergistic micronutrients such as selenium, zinc, and vitamin D—likely amplified thyroid dysfunction risks. For instance, selenium deficiency reduces glutathione peroxidase activity, exacerbating oxidative damage to thyrocytes, while vitamin D insufficiency may heighten susceptibility to autoimmune thyroid diseases via immunomodulatory pathways (29). Our findings of elevated TgAb levels in childhood-exposed individuals further suggest that early-life malnutrition increases autoimmune thyroid disorder risks. Notably, FT3 levels demonstrated an inverse association with childhood famine exposure. However, deficient thyroid hormone secretion may promote thyroid cell hyperplasia, thereby increasing the risk of nodule formation (30).

These mechanisms collectively drive a dynamic interplay between compensatory adaptation and thyroid dysfunction in famine-exposed populations amid improved iodine nutrition. While the USI policy markedly reduced goiter prevalence, thyroid nodules and autoimmune thyroiditis incidence remain unchanged (31), potentially reflecting complex interactions between developmental programming and environmental interventions.

Our study is the first to reveal that famine exposure during different developmental stages differentially impacts thyroid disease risk in later life. However, several limitations should be acknowledged. First, as a cross-sectional study with potential exposure misclassification due to the lack of precise nationwide famine start/end dates, we cannot establish causality between early-life famine exposure and adulthood thyroid disorders. Nevertheless, our findings demonstrate distinct effects of famine timing on thyroid function and disease risk, highlighting the necessity of stage-specific nutritional interventions for thyroid disease prevention and providing a foundation for future cohort studies. Second, participants were recruited from a hospital-based health examination center, a population that typically exhibits greater health awareness and healthier lifestyles than the general community. This may lead to an underestimation of the true effects of early-life famine exposure. To mitigate this potential selection bias, we adjusted for key confounders, including smoking history, alcohol consumption, dietary salt preference, physical activity, and education level. Third, while iodine intake significantly influences thyroid function, our retrospective analysis lacked direct measurements. However, since all participants were from the same region with presumably similar iodine intake, and dietary preferences were adjusted for in our models, the findings remain credible.

## Conclusion

In summary, this study demonstrates that compared to childhood famine exposure, fetal famine exposure was significantly associated with reduced risk of thyroid nodules in later life. After FDR correction for multiple comparisons, fetal exposure showed a suggestive but non-significant association with hyperthyroidism. Additionally, childhood famine exposure significantly increases adult TgAb levels. These findings highlight the critical role of developmental timing in the nutritional programming of thyroid health, suggesting that differences in the “critical window period” of nutritional interventions may influence the spectrum of adult thyroid diseases through distinct mechanisms. This provides important insights for understanding thyroid disease prevention and control during current nutritional transitions.

## Data Availability

The raw data supporting the conclusions of this article will be made available by the authors, without undue reservation.
